# Recent advances in the genetics and innate immune cells of bullous pemphigoid

**DOI:** 10.3389/fimmu.2025.1530407

**Published:** 2025-06-18

**Authors:** Xiaoli Yang, Panling Wei, Zaixing Wang

**Affiliations:** ^1^ Department of Dermatology, The First Affiliated Hospital of Anhui Medical University, Hefei, Anhui, China; ^2^ Institute of Dermatology, Anhui Medical University, Hefei, Anhui, China; ^3^ Key Laboratory of Dermatology (Anhui Medical University), Ministry of Education, Hefei, Anhui, China

**Keywords:** bullous pemphigoid, genetics, genetic susceptibility, innate immune cells, immune cells

## Abstract

Bullous pemphigoid (BP) is a common autoimmune subepidermal blistering disease that primarily affects elderly patients. The pathogenesis of BP is complex, involving genetic, immune, and environmental factors. Recent evidence suggests that multiple genomic regions, particularly within the human leukocyte antigen (HLA)-II region, influence susceptibility to BP. Genetically predisposed individuals may carry susceptibility alleles that modulate the immune system, leading to an elevated risk of developing BP when exposed to the appropriate environmental triggers. Here, the present review discusses the genetics of BP and the critical role of the innate immune system in BP pathogenesis, focusing on the composition of innate immune cells.

## 1 Introduction

Bullous pemphigoid (BP) is the most common autoimmune subepidermal blistering disease affecting the skin and mucous membranes, primarily occurring in elderly patients and characterized by recurrent bullous lesions on the trunk and limbs. The global incidence of BP is approximately 0.0419 cases per 1,000 person-years, with an average clinical prevalence of approximately 0.79%. Compared to patients aged 60–69 years, those over 80 years old have up to a six-fold increased risk of developing BP (1). BP is characterized by circulating IgG autoantibodies against the BP180 (also known as BPAG2) and BP230 (also known as BPAG1) structural proteins within the epidermal basement membrane zone, resulting in the separation of the epidermis from the dermis (2). In patients with BP, the BP180 and BP230 antigens are absorbed and processed by antigen-presenting cells (APCs), where they bind to major histocompatibility complex (MHC) class II molecules before being exposed on the cell surface. The histopathological features of BP include subepidermal blisters and moderate to dense inflammatory infiltrates composed of lymphocytes, neutrophils, and eosinophils (3). The most common cause of death in BP patients is opportunistic infections resulting from prolonged iatrogenic immunosuppression (4). Currently, the exact etiology of BP remains unclear, and neurological diseases (such as multiple sclerosis) (5), psychiatric disorders (6), inflammatory skin conditions (7), and diseases associated with being bedridden are considered risk factors for BP (8). Various factors, including trauma, burns (9), radiation therapy (10), and medications (11), may be involved in the development of BP.

The pathogenesis of BP depends on the interactions among triggering factors (such as human leukocyte antigen [HLA] genes), comorbidities, aging, and environmental triggers (12). Moreover, age-dependent changes impact both adaptive and innate immune responses (13). As BP patients age, macrophage activation and function decrease (14), and neutrophil chemotaxis and phagocytic capacity are reduced (15).Aging negatively affects the chemotaxis, endocytosis, and migratory abilities of dendritic cells (DCs), which in turn exacerbates the severity of BP (16). Although BP is not a typical hereditary disease and its heritability is not well understood, genetic studies have indicated that individuals with certain HLA alleles have a higher risk of developing specific autoimmune blistering diseases compared to those without these alleles (17). Genetically susceptible individuals may carry predisposition alleles that modulate the immune system, leading to an elevated risk of developing BP when exposed to appropriate environmental triggers (18). The present review discusses the genetics of BP and the role of innate immune cells in the pathogenesis of BP.

## 2 Association of HLA with BP

### 2.1 Association between class I HLA and BP

The major histocompatibility complex (MHC) is a highly polymorphic region located on the short arm of chromosome 6 (6p21) and is an essential component of the immune response. The MHC is considered the region most associated with human diseases in the genome. The MHC genomic region is also known as the HLA region in humans. HLA antigens are divided into three classes, namely, HLA class I (HLA-A, -B, and -C), HLA class II (HLA-DR, -DQ, and -DP), and HLA class III (including complement and cytokine genes) (19, 20). HLA-G is a non-classical HLA class I molecule that regulates the balance between Th1 and Th2 cells (21). Recent studies have suggested that HLA class I genes may be closely related to autoimmune diseases, as they are involved in processing and presenting peptides for T cell recognition and are also associated with transplant compatibility. Fang et al. (22) were the first to report that the HLA-A11:01, -B37:01, and HLA-G*0101, 0106 alleles are associated with BP susceptibility in the northern Han Chinese population. Chagury et al. (23) reported that the HLA-C*17 allele is associated with the development of BP in the Brazilian population. However, Christian et al. (24) tested the HLA-Cw6 allele in 40 pemphigus vulgaris patients and 40 BP patients; they detected the HLA-Cw6 polymorphism in 4 out of 40 BP patients, and they reported an HLA-Cw6 genotype frequency of 10% in the BP group but without statistically significant differences between the groups. Banfield et al. (25) and Schaller et al. (26) also reported no significant association between HLA class I genes and BP in the British Caucasian population. The conflicting conclusions may stem from variations in geographic location and population differences in race and ethnicity linked to genetic risk factors. Additionally, confounding variables, such as socioeconomic status, access to healthcare, and the impact of specific environmental factors, may significantly contribute to the various conclusions (27, 28). Future studies are essential to further elucidate the association between HLA class I genes and BP.

### 2.2 Association between HLA-DR and BP

Most studies have reported the widespread presence of HLA lass II genes in BP patients, with susceptibility showing ethnic differences. Seignalet et al. (29) were the first to report the relationship between HLA and BP in French patients, identifying a potential association between HLA-DR5 and BP. Subsequently, Okazaki et al. (30) reported an association of the HLA-DRB1*04 (0403, 0406) and DRB1*1101 alleles with BP susceptibility in Japanese patients. However, a small-sample study conducted in the Chinese population has demonstrated that the HLA-DRB108 allele has a protective effect against BP. Recently, Fang et al. (22) also reported that in the Han Chinese population, the HLA-DRB1*07:01 allele has a protective effect against BP, while the HLA DRB1*10:01 allele is associated with BP susceptibility. The first genome-wide association study (GWAS) conducted in Germany has revealed that the HLA-DRB1*07:01 and HLA-DQA1*05:05 loci have the strongest association with BP (31). Andreani et al. (32) analyzed 30 cases of idiopathic BP and 86 cases of dipeptidyl peptidase-4 inhibitor (DPP4i, such as gliptin drugs)-related BP, and they identified a significant association of the Italian BP population with the DRB1*11:01, DRB1*11:04, and DRB3*02:02 alleles.

### 2.3 Association between HLA-DQ and BP

#### 2.3.1 Distribution characteristics of HLA-DQ alleles across ethnic populations

In addition to HLA-DR, HLA-DQ is also highly prevalent in BP patients, with HLA-DQB1 being particularly important in BP susceptibility. Delgado et al. (33) were the first to identify a significant association between HLA-DQB1*03:01 and all clinical types of pemphigus (BP, oral pemphigus, and ocular scarring pemphigus) in the Caucasian population. Iranian BP patients exhibit genetic susceptibility associated with HLA-DQB1*03:01, similar to that observed in the Caucasian population (34). Subsequently, a significant association has also been reported in the BP population in Brazil (23). Of note, some studies have reported that this association may be more pronounced in male BP patients, though the underlying mechanisms remain to be elucidated (25). A cohort study in Japan has revealed that BP patients have a significantly higher frequency of the DQB1*0302 allele compared to the control group. In a small sample study of the Chinese population, Gao et al. (35) was the first to demonstrate that the DRB1*08/DQB1*06 (DR8/DQ6) haplotype has a protective effect against BP. Subsequently, in a cohort study of 105 BP patients, Fang et al. (22) reported that the frequency of the HLA-DRB1*13-DQA1*05-DQB1*03 haplotype is significantly higher in BP patients compared to the control group. A meta-analysis has revealed that HLA-DQA1*0505 is associated with an increased risk of BP, while DQA1*0201 may have a protective effect against BP (36). In a recent study involving 572 BP patients and 976 healthy controls, Sun et al. (17) reported that DQB1*03:01 is the only significant risk association for BP in the Chinese population, while DQB1*03:03 and DQB1*06:01 are significant protective associations. Further stratified analysis demonstrated that while DQB1*03:01 showed significant associations with both BPAG1-positive (*P*=5.65×10^−3^, OR=1.371) and BPAG2-positive (*P*=7.99×10^−8^, OR=1.638) patients, it exhibited a stronger risk effect in the BPAG2 group. This difference suggests that DQB1*03:01 may preferentially participate in BP180-mediated autoimmune responses. The underlying mechanism may involve the HLA protein encoded by DQB1*03:01, which can bind multiple T-cell epitopes within both BP230 and BP180 but may possess higher binding affinity or immunogenicity for BP180 epitopes (37).

#### 2.3.2 Association mechanisms between HLA-DQ and neurological comorbidities/DPP4i-associated BP

A recent meta-analysis has reported that BP patients are five times more likely to develop neurological diseases (NDs) compared to the general population (38). BP is associated with various NDs, such as dementia, stroke, Parkinson’s disease, epilepsy, multiple sclerosis, and polyneuropathy. The coexistence of BP and NDs is independently associated with the production of anti-BP230 antibodies (39). Murali et al. (40) demonstrated that the DRB1*04-DQB1*0301, DRB1*07-DQB1*02, DRB1*07-DQB1*0301, DRB1*11-DQB1*0301, and DRB1*13-DQB1*06 haplotypes are strongly associated with susceptibility to ischemic stroke in the South Indian population. The interaction between HLA-DQB1*0301 and DQB1*0302 is also associated with multiple sclerosis in European patients (41). The presence of the HLA-DQB1*03:01 allele may provide a link between the development of NDs and BP. Future GWAS and next-generation sequencing studies are crucial for further investigating the HLA profiles of BP patients with comorbid NDs, which will help better understand the genetic mechanisms underlying the association between BP and NDs. This research should not only involve thorough HLA typing of BP patients with NDs but also include epitope mapping to confirm that the presence of specific antibodies is related to HLA-binding sites (42). In addition, some studies have reported that oral DPP4i are one of the factors contributing to an increased incidence of BP in patients with type 2 diabetes (43). DPP4i exposure is associated with more than a threefold increased risk of developing BP (44). Ujiie et al. (45) conducted a study on 30 Japanese patients with DPP4i-related BP and reported that 86% (18/21) of the non-inflammatory DPP4i-BP patients carry the HLA-DQB1*03:01 allele, suggesting that HLA-DQB1*03:01 may be a useful biomarker for predicting DPP4i-related BP in Japanese patients before treatment. In a study on 100 Thai BP patients, Chanprapaph et al. (46) reported that the presence of HLA-DQB1*03:01 is significantly more frequent in DPP4i-related BP patients compared to diabetic BP cases without DPP4i exposure and those who tolerated DPP4i. Consistent with findings in Japanese patients, HLA-DQB1*03:01 may serve as a useful biomarker for predicting DPP4i-related BP in Thai patients before treatment. A recent study in the Italian population has also reported a significant association of the HLA-DQB1*03:01 allele with both idiopathic BP and DPP4i-related BP patients (32). In contrast, a cohort study conducted in Finland has reported no significant difference in the frequency of the DQB1*03:01 allele in BP patients treated with DPP4i or untreated BP patients compared to healthy controls (47).

#### 2.3.3 Challenges and future directions in HLA-DQ research

The contradictory findings in these studies, aside from ethnic differences in susceptibility, should particularly focus on the impact of linkage disequilibrium (LD) within the HLA gene region (48). Due to the complex genetic structure and high LD in the HLA region, apparent associations between certain HLA loci and BP may be driven by other functional loci in strong LD with them (49). Notably, there is a significant LD between the HLA-DR and HLA-DQ genes, which is evident in European, Middle Eastern, and Asian populations (28). When conducting association studies of HLA-DR, HLA-DQ, and BP, it is crucial to consider the LD effects within the HLA region, as different populations may exhibit distinct LD patterns (50). Recently, a GWAS study by Ozeki et al. (51) demonstrated that DPP4i-induced noninflammatory BP could be explained by HLA-DQA1∗05 and/or HLA-DQB1∗03:01, which are in strong LD with each other. Moreover, there was no associated SNP in conventional BP in contrast to that in DPP4i−induced noninflammatory BP, indicating that these clinical variants may have different genetic mechanisms. Future studies should adopt more advanced genomic profiling techniques and statistical methods to correct for the impact of LD. Additionally, confounding variables, such as socioeconomic status, access to healthcare, and specific environmental factors, should be controlled to further clarify the relationship between HLA genes and BP.

##### 2.3.3.1 Non-HLA regions and their association with BP

In addition to the HLA region, gene polymorphisms in low-affinity Fcg receptors (FcgRs), the mitochondrial ATP synthase 8 gene (MT-ATP8), ABCB1, cytokines, and other genes may also influence susceptibility to BP (52). Low-affinity FcgRs, which are receptors for the Fc region of IgG, are associated with various autoimmune diseases, and they play a role in regulating the interaction between antibodies and inflammatory cells, acting as a bridge between specific antibodies and effector cells, thereby linking humoral immunity and cellular immunity. Andreas et al. reported that both allele and copy number variations (CNVs) of FcgR genes affect the mRNA expression of granulocyte FcgR and the release of reactive oxygen species, which further leads to pathogenicity mediated by autoantibodies. These findings suggest that CNVs in FcgR IIc gene gain and FcgR IIIb gene loss are genetic susceptibility factors for BP (53). The ABCB1 gene encodes the membrane transport protein P-glycoprotein. Some studies have found that genetic variations in ABCB1, such as the G2677 T/A polymorphism, may be associated with the development of BP (54). In addition, genetic polymorphisms in cytokines may influence their function by regulating the expression and release of corresponding cytokine proteins, thereby regulating the susceptibility to autoimmune diseases. In BP patients, the expression of various cytokines is elevated in blister fluid, serum, or skin tissue, particularly Th17- and Th2-associated cytokines. Previous studies in China have found that BP is associated with polymorphisms in female interleukin (IL)-1β at positions -511 and -31 (55). Subsequently, Tabatabaei-Panah et al. investigated single nucleotide polymorphisms (SNPs) in several cytokines, including IL-1α (rs1800587), IL-1β (rs1143627, rs16944, and rs1143634), IL-8 (rs4073), and TNF-α (rs1799964, rs1800630, rs1799724, and rs361525) in BP patients and healthy controls, as well as IL-8 (rs4073) in patients with pemphigus vulgaris; compared to IL-1α, IL-1β, and TNF-α, the expression level of IL-8 (rs4073) is significantly higher in BP patients than in healthy controls, suggesting that the minor allele in the IL-8 SNP may play a protective role in the susceptibility of Iranian patients to BP (56). Wang et al. conducted serum level and SNP analysis of multiple cytokines in 61 BP patients; they reported that BP is associated with gene polymorphisms in cytokines, such as IL-13, IL-1β, TNF-α, and IFN-γ, and they also observed a significant association between IL-13 and the tendency for BP recurrence. Specifically, the genotypes at the rs20541 and rs1800925 loci of IL-13 are related to sex. These findings suggest that IL-13 is involved in the pathogenesis of BP and may serve as a potential therapeutic target and prognostic marker for BP treatment (57, 58). Tabatabaei-Panah et al. suggested that the alleles of two SNPs in IL-23R (rs2201841 and rs7530511) are associated with BP (59).

##### 2.3.3.2 Genes related to innate immunity

Classical HLA class I and II genes play a pivotal role in T cell-mediated adaptive immunity by facilitating the recognition of antigenic peptides through T cell receptors (TCRs). These antigenic peptides, presented by HLA molecules on the surface of APCs, initiate T cell responses. HLA class I molecules interact with CD8+ T cells, while HLA class II molecules primarily engage CD4+ T cells, enabling precise recognition of HLA-peptide complexes (19). HLA class II genes, predominantly expressed on professional APCs, bind peptides derived from extracellular antigens and present them to CD4+ T cells. This recognition activates the regulatory helper functions of CD4+ T cells, promoting their differentiation into TH1 or TH2 phenotypes and orchestrating the broader immune response (60). CD4+ T cells play a central role in activating the immune response in BP. Based on the type of inflammatory response, CD4+ T cells differentiate into various subsets, including Th1, Th2, Th17, follicular helper T cells (Tfh), and regulatory T cells (Tregs) (61). HLA-G is a non-classical HLA class I molecule that plays a role in modulating the balance between Th1 and Th2 cells (21). Both self-reactive Th1 and Th2 cells may be involved in regulating the production of pathogenic autoantibodies by B cells in BP patients (62). These BP autoantibodies contribute to an inflammatory response by inducing the migration of a large number of eosinophils and a smaller number of neutrophils into the dermis, where they degranulate. These inflammatory cells contain and release various cytokines, chemokines, hydrolytic enzymes (including matrix metalloproteinase 9 [MMP-9] and neutrophil elastase [NE]), and reactive oxygen species (ROS) upon activation. This cascade of inflammation ultimately leads to tissue damage and the formation of subepidermal blisters (63).

**Figure 1:**
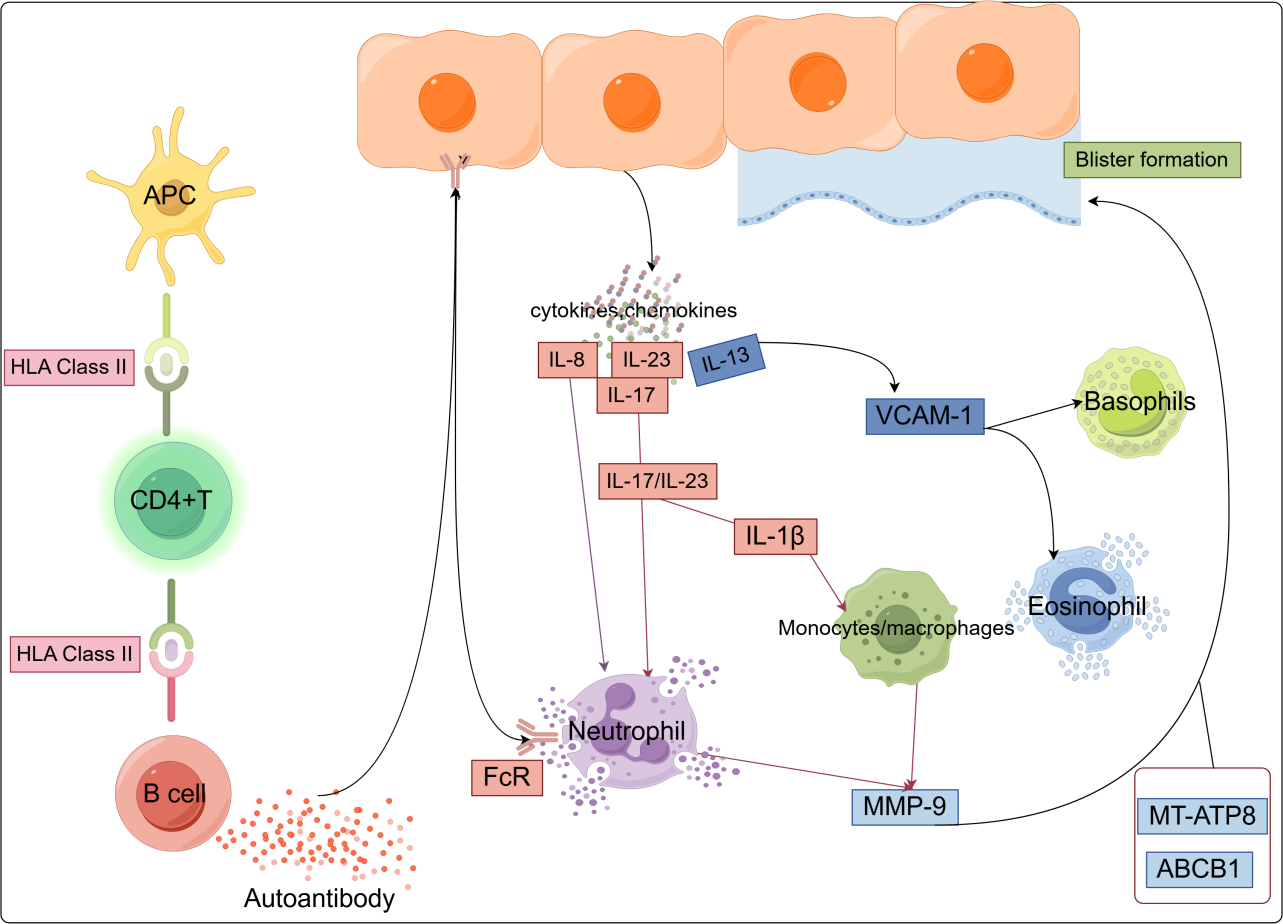
Major pathways and innate immune cell types associated with BP, illustrating the pathways in which BP susceptibility genes may function within innate immune cells.

In BP patients, the expression of various cytokines is elevated in blister fluid, serum, or skin tissue, particularly Th17- and Th2-related cytokines. Th17 cells promote autoimmune pathology by secreting IL-17, IL-21, IL-22, IFN-γ, and granulocyte-macrophage colony-stimulating factor (GM-CSF). Th2 cells secrete IL-4 and IL-13, with IL-13 specifically promoting the recruitment of eosinophils to sites of allergic inflammation (64). As mentioned earlier, genetic polymorphisms of IL-1β, IL-13 (rs20541 and rs1800925), IL-8 (rs4073), and IL-23R (rs2201841 and rs7530511) influence the susceptibility to BP. IL-13 upregulates the gene expression of vascular cell adhesion molecule-1 (VCAM-1), which is a cell adhesion molecule and a marker of endothelial activation. VCAM-1 plays a crucial role in the migration of eosinophils and basophils, which express VLA-4, a VCAM-1 ligand (65). IL-13 activates eosinophils to secrete various cytokines, including IL-13 itself, which further promotes the maturation of B cells into plasma cells and mediates the secretion of IgE antibodies by plasma cells. Additionally, IL-13 converts Th0 cells into Th2 cells, helping to sustain and amplify the autoimmune process (57). Zhang et al. used single-cell RNA sequencing and *in vitro* functional analysis to identify Th2 cells, DCs, and fibroblasts as key cell populations involved in BP. The IL13-IL13RA1 ligand-receptor interaction has been reported to be the most important mediator of the immune-matrix crosstalk in BP. Additionally, fibroblasts and DCs expressing IL13RA1 respond to IL13-secreting Th2 cells. This response is amplified by the specific upregulation of PLA2G2A in fibroblasts and CCL17 in myeloid cells, creating a positive feedback loop that enhances the Th2-mediated cascade and promotes immune-matrix crosstalk. These findings reveal the mechanisms controlling immune-matrix interactions and provide potential therapeutic avenues for future research (66). Additionally, IL-23R amplifies the Th17 cell response by inducing proinflammatory cytokines and dysregulated IL-23 production, thereby promoting autoimmune inflammation (59). IL-23 is an upstream cytokine of IL-17, and the IL-17/IL-23 axis increases MMP-9 secretion in monocytes and neutrophils, promoting blister formation (67, 68) (**Figure 1**).

Genetic evidence confirms novel insights into critical immune pathways and supports the recruitment and activation of innate immune cells (such as DCs, monocytes/macrophages, neutrophils, eosinophils, and basophils) as playing a key role in BP (63). Current evidence indicates that the pathogenesis of BP involves a complex network of diverse immune cells and cytokines, suggesting that inhibiting aberrant activation of innate immune cells may represent a critical intervention point for preventing disease onset (68, 69).However, the precise regulatory mechanisms underlying innate immune cell activation in the context of genetic predisposition during BP progression remain unclear. In the following sections, we will discuss in detail the role of innate immune cells in BP pathogenesis by integrating the latest genetic evidence.

## 3 Role of innate immune cells in the pathogenesis of BP

### 3.1 DCs

#### 3.1.1 Antigen presentation by DCs drives the autoimmune response in BP

HLA class II antigens are expressed only on certain immune-active cells and play a crucial role in mediating various immune functions, including antigen presentation to T cells and target recognition by cytotoxic T cells. In normal epidermis, only Langerhans cells (LCs) express HLA-D antigens (70, 71). LCs are antigen-presenting and myeloid-derived immature DCs found in the epidermis, and they are the first cells to encounter skin pathogens. Under various stimuli, LCs are activated into mature DCs. IL-18 induces the migration of LCs and the accumulation of DCs. Mature LCs migrate to skin-draining lymph nodes, where they present antigens to CD4+ T cells, thereby regulating adaptive immune responses (72, 73). DCs, as potent APCs, play a pivotal role as a bridge between innate and adaptive immunity. DCs initiate innate immune responses and process antigens by recognizing pathogen-associated molecular patterns through pattern recognition receptors, such as Toll-like receptors. Additionally, DCs activate naïve T cells and direct the differentiation of effector T cell subsets through antigen presentation and the expression of co-stimulatory molecules (e.g., CD34). This dual functionality is crucial for the initiation and amplification of adaptive immunity (74, 75). As a result, DCs are indispensable in the pathogenesis of various autoimmune diseases, including systemic lupus erythematosus (76), rheumatoid arthritis (77), multiple sclerosis (78), and psoriasis (79). Compared to healthy adults, skin lesions in BP contain a higher number of LCs and DCs, which may process basement membrane zone (BMZ) antigens (80). Li et al. (81) reported a high number of DC-specific intercellular adhesion molecule-3-capture integrin (DC-SIGN)-positive DCs in the skin lesions of BP patients, suggesting that DC-SIGN-positive DCs may be involved in the pathogenesis of BP. Moreover, the expression of thymic stromal lymphopoietin (TSLP) is significantly upregulated in DC-SIGN-positive cells, and most of these cells express the TSLP receptor. Thus, TSLP may directly activate DC-SIGN-positive DCs to participate in the development of BP. However, further research is needed to identify the specific subsets of DCs activated by TSLP, clarify the relationship between LCs and TSLP, and explore the downstream mechanisms of TSLP and DCs.

#### 3.1.2 Synergistic interactions between DCs and microenvironmental cells

**Figure 2:**
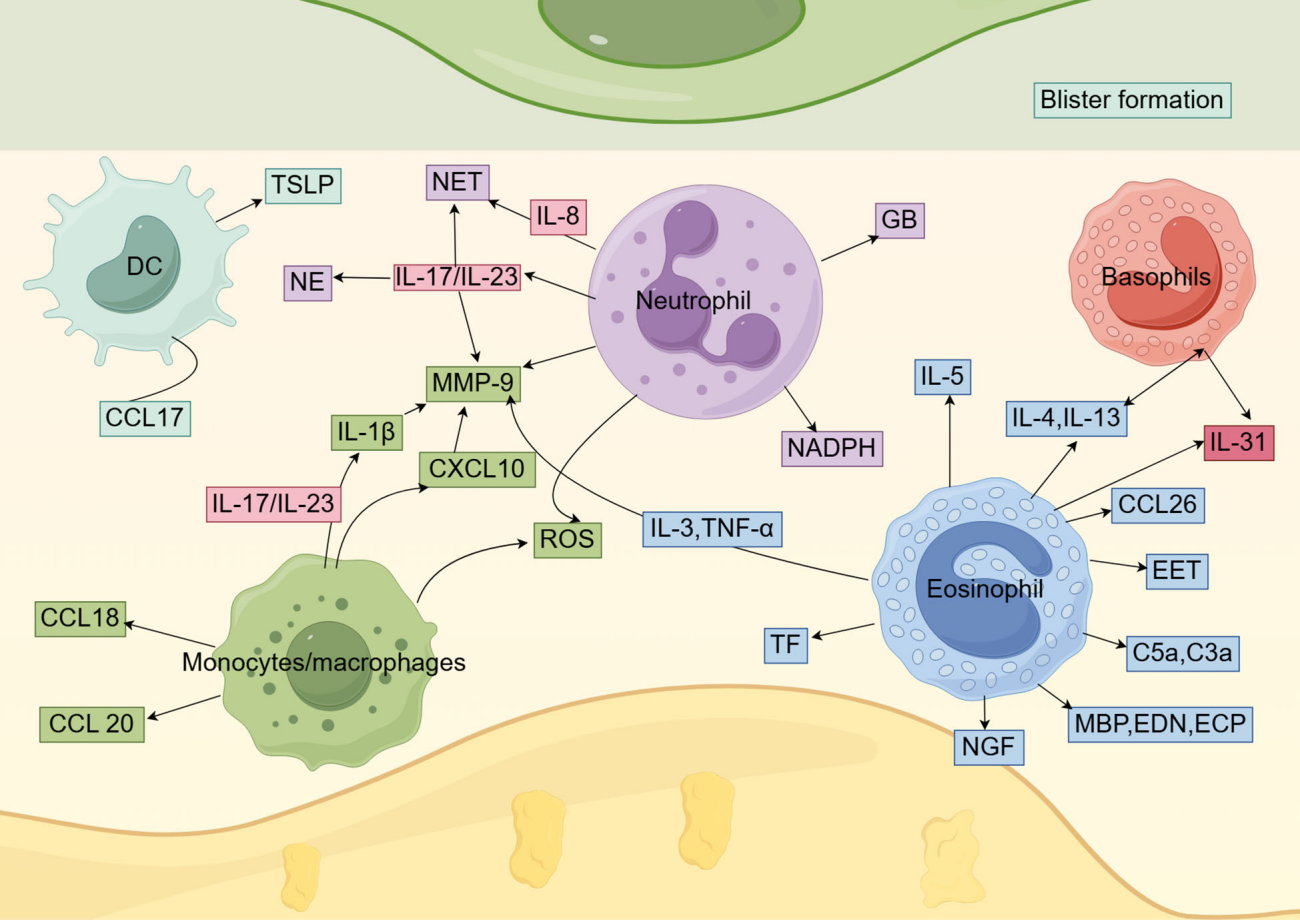
The role of innate immune cells in BP. Dendritic cells, monocytes/macrophages, neutrophils, eosinophils, and basophils play a role in the pathogenesis of BP by secreting a variety of cytokines and chemokines.

Recent studies have also found that fibroblast-derived PLA2G2A may drive the secretion of CC chemokine ligand 17 (CCL17) by myeloid cells, particularly DC clusters, in BP patients. Treatment with recombinant CCL17 significantly increases IL-13 secretion by peripheral blood mononuclear cells (PBMCs) in BP patients and increases the titer of anti-BP180-NC16A autoantibodies in BP PBMCs, further elucidating the immune mechanisms underlying BP (**Figure 2**) (66). In summary, DCs play a pivotal role in the pathogenesis of BP through antigen presentation and immune regulation. Future studies should further elucidate the precise mechanisms of DCs in BP development, which may provide novel therapeutic targets for BP treatment.

### 3.2 Monocytes and macrophages

#### 3.2.1 Phenotypic polarization and functional characteristics of macrophages

Macrophages exhibit significant plasticity, allowing them to alter their physiological functions in response to environmental factors. Macrophages can adopt an M1 phenotype, which is associated with inflammatory or classical activation, or they can adopt an M2 phenotype, which is characterized by anti-inflammatory or alternative activation (82). M1 macrophages produce high levels of proinflammatory molecules, such as TNF-α, IL-1, IL-6, IL-23, IL-12, type I interferons (IFNs), reactive nitrogen intermediates, reactive oxygen intermediates, CXCL9, CXCL10, and CXCL11 (83). In contrast, M2 macrophages express IL-4, IL-10, CD163, and CD206, and they promote tissue regeneration and repair (84, 85). By single-cell analysis, Ruan et al. (86) identified a significant increase in the proportion of myeloid compartment NLRP3[+] and C1q[+] macrophages in BP lesions compared to healthy skin and atopic dermatitis lesions, suggesting a potential role of macrophages in the pathogenesis of BP. NLRP3^+^ macrophages in BP lesions actively recruit various immune cells, including neutrophils and Tph-like Th2 cells. These Tph-like Th2 cells are characterized by high PD-1 expression but lack BCL6/CXCR5. By secreting key cytokines such as IL-21 and CXCL13, they not only mediate type 2 immune responses but also specifically promote B cell recruitment and antibody production (87). C1q[+] macrophages in BP lesions exhibit unique transcriptional features, characterized by enhanced expression of chemokines, such as CCL13 and CCL24, which play crucial roles in the recruitment of T cells and basophils, respectively (88–90).

#### 3.2.2 Core pathogenic mechanisms mediated by macrophages

Macrophages are also critical for subepidermal blister formation in BP mouse models, potentially through the recruitment of neutrophils (91). In BP blister fluid, MMP-9 is one of the key proteases involved in the pathological process. These proteases degrade extracellular matrix proteins and BP180, resulting in dermal-epidermal separation and blister formation (92–94). MMP-9 is also a crucial molecule in the infiltration of inflammatory cells (95). As mentioned earlier, IL-1β and IL-23R are linked to BP susceptibility. Le Jan et al. (96) reported that the IL-23/IL-17 axis activates inflammasomes in macrophages, leading to the release of IL-1β. IL-1β, in turn, induces MMP-9 secretion in monocyte-derived macrophages. Therefore, IL-1β released by macrophages may contribute to the amplification of the self-sustaining inflammatory cycle in BP-associated spontaneous inflammation. Chakievska et al. (97) also reported that IL-17 in blister fluid induces the production of M2-polarized macrophages, which produce MMP-9 and contribute to the development of skin lesions in BP patients. Notably, regarding the mechanisms underlying BP development, Ohuchi et al. suggested that the CXCL13/CXCR5/anti-BP180-NC16A antibody axis may be initiated by the IL-17/IL-37 pathway in macrophages. In addition, the overexpression of IL-17RA and IL-17RC in monocytes may play a role in BP recurrence, as these receptors are downregulated in BP patients with controlled disease but remain overexpressed in those with recurrent BP (98). Additionally, *in vitro* stimulation of CD163[+] M2 macrophages with LL37 induces the production of CXCL10 and CCL20 (99). Notably, CXCL10 not only enhances IL-17 expression in monocytes/lymphocytes of BP patients through autocrine and paracrine pathways, forming an inflammatory amplification loop, but also specifically promotes the secretion of MMP-9 by monocytes and neutrophils (90). In BP, M2 macrophages also produce CCL18, which is linked to autoimmune diseases (100, 101). Additionally, serum levels of CCL18 are elevated in BP patients (**Figure 2**) (102). Current literature on macrophages primarily focuses on the M2 subtype, while the role of the M1 subtype in BP remains largely unexplored. Additional research is needed to further clarify the molecular mechanisms and regulatory factors of macrophages in BP and to develop effective drugs targeting these pathways (69). Specifically, the development of specific inhibitors targeting the NLRP3/IL-1β/MMP-9 axis may hold significant therapeutic potential.

### 3.3 Neutrophils

Neutrophils are the frontline responders in the acute innate immune response to invading pathogens and are the most abundant effector cells in the human immune system. Neutrophils play a crucial role in maintaining homeostasis and supporting immune responses within the body (103). In BP, neutrophils are one of the first inflammatory cell types to infiltrate skin lesions and are key cellular elements in triggering dermal-epidermal separation (104). Neutrophils are critical effector cells in the pathogenesis of blister formation in experimental BP models. Subepidermal blistering in these models is mediated by the interaction between the Fc region of anti-BP180 IgG and the Fc receptors on neutrophils. As previously mentioned, a high copy number of FcgRIIc and a low copy number of FcgRIIIb may be associated with increased susceptibility to BP (53). FcgRIII, in particular, is a key receptor that facilitates the binding of pathogenic anti-mBP180 IgG and activates infiltrating neutrophils (105). This process is also dependent on the activity of NE (104). NE is an enzyme secreted by the first wave of activated neutrophils that cleaves mBP180 within the immunodominant NC14A domain, generating a 12-kD degradation product known as p561, which exhibits chemotactic effects on neutrophils, both *in vitro* and *in vivo (* 106). NE activity is crucial for the formation of subepidermal blisters in experimental BP. NE acts alone or in combination with other effector molecules to degrade components of the BMZ that maintain dermal-epidermal cohesion, thereby playing a pathogenic role in BP (107). In addition to NE, neutrophil granules contain several proteolytic enzymes, including cathepsin G (CG), collagenase, and gelatinase B (GB). GB released by neutrophils plays a key role in subepidermal blister formation in experimental BP. GB can directly cause tissue damage in BP by cleaving structural proteins at the dermal-epidermal junction or indirectly contribute to tissue damage by inactivating major neutrophil elastase inhibitors, such as α1-PI, or other neutrophil-derived protease inhibitors. These enzymes may also play a role in the long-term progression of the disease (108). As previously mentioned, CXCL10 produced by macrophages stimulates neutrophils to secrete MMP-9, thereby potentially driving an inflammatory loop that is associated with disease outcomes in BP patients (90). In contrast, neutrophil-produced ROS and neutrophil-derived nicotinamide adenine dinucleotide phosphate (NADPH) oxidase have been shown to play a crucial role in tissue damage induced by autoantibodies in a BP cryosection model (109). ROS and reactive nitrogen species, key redox molecules in immunity, are produced by activated neutrophils and monocytes through NADPH oxidase and NOS. Neutrophils produce higher levels of ROS, while monocytes generate more reactive nitrogen species (110). Monocytes also promote neutrophil recruitment and ROS production, leading to significantly increased dermal-epidermal separation (111). Additionally, we previously reported that IL-8 (rs4073) affects BP susceptibility. Neutrophils release neutrophil extracellular traps (NETs) through apoptosis. Studies have shown that the number of NETs in the skin lesions, serum, and blister fluid of BP patients is higher than in corresponding samples from healthy controls. Elevated levels of key inflammatory factors, such as IL-8, in BP serum and blister fluid induce NET formation. The BP immune microenvironment contains immune complexes that bind to Fc receptors on neutrophils, inducing NET formation in circulation and skin lesions. These abnormal NETs promote B cell differentiation into plasma cells and enhance the production of autoantibodies by activating the MAPK p38 cascade (112). In addition, the IL-17A and IL-23 cytokines are elevated in the serum of BP patients at risk of disease recurrence. The IL-17/IL-23 axis promotes various pathological processes, including stimulating the production of neutrophil MMP-9 and NE, as well as the release of NETs (**Figure 2**) (97, 113). After IL-17 recruitment and activation, neutrophils produce more IL-17 and release proteases, leading to matrix degradation and the generation of matrix peptides, which further enhance neutrophil recruitment and activation at the site of skin lesions (88). Recent studies have revealed a strong correlation between significant neutrophil infiltration and comorbid psoriasis in BP patients (114). In an ex vivo model using normal human skin cryosections, BP patient IgG-induced separation of the dermal-epidermal junction (DEJ) depends on neutrophils activated by immune complexes (115). In summary, neutrophils play a pivotal role in the pathogenesis of BP through multiple mechanisms, including Fc receptor-mediated antibody interactions, release of proteolytic enzymes, generation of oxidative stress, and activation of cytokine networks. Future studies should further elucidate the precise mechanisms of neutrophil involvement in BP, which may provide novel therapeutic targets for this disease.

### 3.4 Eosinophils

Eosinophil infiltration and peripheral eosinophilia are considered early and key events in the development of BP lesions. Peripheral eosinophilia is observed in 50% to 60% of BP cases and is positively correlated with disease severity (116, 117). Of note, healthy relatives with high-risk HLA class II alleles exhibit T cell responses after treatment with recombinant NC16A. In individuals with BP, a Th2 response is observed, and eosinophils drive Th2 polarization through differential cytokine release (118). As previously mentioned, BP is associated with IL-13 gene polymorphisms. Hashimoto et al. (119) suggested that IL-13 contributes to BP-related pruritus by directly stimulating peripheral nerve fibers and/or indirectly by recruiting eosinophils, which then promote peripheral nerve damage. Compared to patients with normal eosinophil counts and percentages, BP patients with serum eosinophilia tend to be older, with more severe palmar-plantar involvement and a higher proportion of indirect immunofluorescence positivity (120). De Graauw et al. (121) demonstrated that in the presence of BP autoantibodies, IL-5-activated eosinophils cleave the skin at the DEJ, directly causing subepidermal blister formation in BP. In skin lesions, eosinophils are activated to release the major basic protein, eosinophil-derived neurotoxin (EDN), and eosinophil cationic protein (ECP). High concentrations of ECP and EDN are present in the serum and blister fluid of BP patients (122). Intradermal injections of EDN and ECP into guinea pig skin over six weeks leads to ulcerative or crusted lesions accompanied by marked cellular infiltration, indicating that these proteins actively contribute to BP skin pathology (123). Furthermore, Amber et al. (124) suggested that ECP and EDN may promote BP pathogenesis by directly affecting keratinocytes, inducing the expression of BP-related cytokines, chemokines, and MMP-9, as well as impairing cell viability and extracellular matrix adhesion. Tsuda et al. (125) also demonstrated that eosinophil degranulation directly damages basal keratinocytes, leading to separation at the DEJ. Eosinophils express receptors for the C3a and C5a complement anaphylatoxins, which not only regulate eosinophil migration but also trigger degranulation. Previous studies have suggested that complement activation plays a critical role in BP blister formation (126). Eosinophils are also key contributors to the secretion of MMP-9 (127). IL-3 and TNF-α stimulate eosinophils to produce large amounts of MMP-9 (128). In a mouse model, MMP-9 has been reported to regulate NE activity by inactivating α1-proteinase inhibitor, leading to further degradation of BP180 and separation of the DEJ (107). Eosinophils are also an important source of tissue factor (TF) in the vasculature, serving as initiators of the extrinsic coagulation pathway. Eosinophils play a role in coagulation activation through TF (129). Tedeschi et al. reported that the levels of ECP in the blister fluid of BP patients are significantly elevated and correlated with coagulation activation markers, leading to inflammation, tissue damage, blister formation, and a potential risk of thrombosis (130, 131). Additionally, ex vivo experiments using human skin and isolated human eosinophils have indicated that eosinophil extracellular traps (EETs) may contribute to DEJ separation, as DEJ separation is significantly reduced after DNase treatment (132). These extracellular traps have been identified in human biopsy skin samples from a range of diseases, including BP (127). Eosinophils also participate in the pathogenesis of BP by mediating the effects of anti-BP180 IgE antibodies and promoting dermal-epidermal separation. When anti-NC16A IgE is introduced *in vivo*, eosinophils are essential for inducing BMZ separation (127, 133). Conversely, Gounni Abdelilah et al. (134) reported that the levels of eosinophil chemotactic factors and MCP-4 are elevated in tissues and blister fluid of BP patients and may be secreted by eosinophils upon stimulation by IgG, IgA, or IgE immune complexes induced by IL-5. An important autocrine pathway may be involved in the recruitment and activation of local eosinophils in BP. In addition to CCL11, Günther et al. (135) linked the upregulation of the eosinophil chemotactic factor CCL26 in BP to the accumulation of activated eosinophils in skin lesions. This finding expands the understanding of BP pathogenesis and may suggest new options for therapeutic intervention. Eosinophils are the major source of IL-31 in BP, and this cytokine may contribute to pruritus in BP patients (136). Importantly, eosinophils from BP patients release higher levels of IL-31 compared to those from healthy donors (**Figure 2**) (137). Elevated serum IL-31 levels in BP patients have also been shown to correlate with peripheral eosinophil counts and the presence of anti-BP180 IgE antibodies. Substance P induces eosinophils to release nerve growth factor (NGF) and IL-31. Due to its ability to sensitize primary pruriceptive neurons, NGF may play an important role in mediating itching (138). In summary, eosinophils play a pivotal role in BP pathogenesis through direct tissue damage and immunomodulatory functions. Their activation involves multiple interconnected pathways, including cytokine networks, the complement system, chemokine axes, and neuro-immune crosstalk. While therapeutic strategies targeting these pathways hold significant clinical potential, further research is needed to elucidate eosinophil heterogeneity and optimize targeting approaches.

### 3.5 Basophils

Basophil infiltration has been observed in various inflammatory skin diseases, including atopic dermatitis, prurigo, and urticaria. This phenomenon has also been observed in autoimmune skin diseases, such as BP (139). However, few studies have investigated the role of basophils in the pathogenesis of BP. In 1982, Dvorak et al. (140) used electron and light microscopy to study the inflammatory response in lesions at different stages of clinical development in BP patients; these researchers were the first to report basophil infiltration in BP skin lesions, primarily found in clinically significant lesions and adjacent normal skin. Moreover, the location of eosinophils within BP lesions is related to their proximity to degranulating basophils. Recently, Hashimoto et al. (119) analyzed skin lesions from 24 BP patients using immunofluorescence staining and reported that the number of basophils infiltrating the dermis is significantly higher in BP skin samples compared to healthy controls. In addition, the number of dermal basophils is significantly correlated with the severity of itching, highlighting the relationship between basophils and BP-associated pruritus. Basophils produce IL-31, which leads to severe itching (141). High levels of IL-31 have been detected in the serum and blister fluid of BP patients. Basophil-secreted IL-31 recruits eosinophils to the lesion site. When recruited eosinophils produce IL-4 and IL-13, more basophils are activated to produce IL-31, thereby forming a positive feedback loop that promotes BP-related pruritus (**Figure 2**) (142). Kimura et al. (143) compared erythematous and bullous lesions in a total of 25 BP patients using histopathology, immunohistochemistry, and electron microscopy, confirming the dual role of basophils in the development and resolution of BP; they reported a positive correlation between the number of basophils and eosinophils in the early stages of BP. During the bullous phase, the number of basophils is significantly higher, but in the later stages, basophils interact with M2 macrophages to inhibit disease progression.

## 4 Impact of immune mechanism advances on the management and treatment of BP

Traditionally, the management of BP has relied on systemic corticosteroids, immunosuppressants, and topical treatments aimed at controlling inflammation and autoimmune responses. However, these approaches have certain limitations, such as significant side effects, prolonged treatment durations, and suboptimal efficacy in some patients (144). This article explores the pathogenesis of BP, the role of innate immune cells, and their interactions with genetic factors. It not only enhances our understanding of BP’s pathophysiology but also lays a crucial foundation for the development of new therapeutic strategies, particularly those targeting innate immune cells and immune molecules. The following section will discuss drugs targeting pathogenic innate immune cells and genetic molecules, along with their current clinical application stages.

### 4.1 Targeted treatment of macrophages and neutrophils

As previously discussed, IL-1β plays a crucial role in BP by inducing the secretion of MMP-9 in macrophages and promoting neutrophil recruitment, degranulation, and NET formation (145). The IL-8 and IL-17/IL-23 axis also influences BP susceptibility and triggers NET formation. Therefore, targeting the IL-1β pathway, particularly the NLRP3 inflammasome, in combination with inhibiting IL-8, the IL-17/IL-23 axis, NE, and MMP, may be a promising approach for BP patients. AC-203 is a topical formulation that modulates the inflammasome and IL-1β pathways. A Phase 2 open-label clinical trial (NCT03286582) has compared 1% AC-203 ointment with 0.05% clobetasol ointment in BP patients, but the trial was terminated in 2019 due to partial recruitment completion (146). DF2156A, an allosteric dual inhibitor of IL-8, was evaluated in a Phase 2 open-label trial (NCT01571895) for its effectiveness in improving BP-related bullous activity; however, the trial was prematurely discontinued, and no further studies have been conducted (147). The potential of targeting IL-17 and IL-23 in BP treatment is still under investigation, and these approaches are primarily used in BP patients with comorbid psoriasis (148). Ixekizumab and secukinumab, both IL-17 inhibitors, have shown efficacy in BP cases complicated by psoriasis (149–151), with secukinumab inducing long-term remission and reducing BP180-NC16A antibody levels (152). Ustekinumab and tildrakizumab, IL-23 inhibitors, have been reported to successfully treat a case of recurrent BP associated with psoriasis (153). However, there are also reports of new-onset BP in some psoriasis patients treated with ustekinumab (154). Kerkemeyer et al. successfully used tildrakizumab to treat refractory lichen planus pemphigoides, but clinical data supporting its use in BP remain insufficient (155). Moreover, Liu et al. (107)demonstrated that NE inhibitors prevent blister formation in NE-deficient and wild-type mice, as well as block DEJ separation in ex vivo human skin models. Williams et al. (156) reported that doxycycline (200 mg/day) effectively controls blisters by inhibiting MMPs and is safer than the standard treatment of oral prednisone (0.5 mg/kg/day). However, NE inhibitors (such as sivelestat) and MMP inhibitors (such as batimastat or andecaliximab) have not yet been used as treatments for BP (157). The exact effects of these treatment options in BP remain uncertain, and larger randomized controlled trials are needed to validate their efficacy and safety.

### 4.2 Targeted treatment of eosinophils and basophils

As previously discussed, some cytokines (IL-5, IL-4, IL-13, and IL-31) and complement factors (C5a and C3a) play crucial roles in the pathogenesis of BP by influencing eosinophils and basophils. Anti-IL-5 monoclonal antibodies, such as mepolizumab and reslizumab, target IL-5 to reduce eosinophil levels in the serum. While mepolizumab does not show an advantage over steroids in clinical trials (158), reslizumab has demonstrated significant effectiveness in treating BP (159).The anti-IL-4/-13 monoclonal antibody, dupilumab, which inhibits IL-4 and IL-13 signaling, has been successfully used in BP treatment, especially when combined with steroids or immunosuppressants, showing superior efficacy compared to steroids alone (160). Dupilumab also targets IL-31 and other pruritus-related signals, offering relief for BP patients with persistent itching (161). Studies have found that dupilumab can not only reduce the dosage of glucocorticoids in BP patients but, most importantly, it is highly safe for elderly patients with multiple comorbidities (162). Complement system inhibitors, such as avdoralimab and nomacopan, which inhibit C5aR1 and C5/LTB4 activity, have shown promising potential in BP treatment, with nomacopan demonstrating significant clinical efficacy (163, 164). Lastly, CD46 represents a novel therapeutic strategy aimed at providing protection against BP pathogenesis by inhibiting C3 deposition, with research supporting its potential clinical application (165). Overall, these immune-modulating antibody therapies offer promising new avenues for BP treatment, though further clinical trials are necessary to confirm their efficacy and safety.

## 5 Conclusions

In summary, BP is an autoimmune disease that primarily affects the elderly, with its susceptibility influenced by both genetic and environmental factors. MHC genes play a central role in mediating the development of the BP immune system, which can become dysregulated when combined with poorly defined environmental triggers, leading to the development of autoantibodies and the onset of the disease pathology. Innate immune cells play a crucial role in driving the activation of the BP immune system. Future research should focus on high-quality integrative technologies, including GWAS and next-generation sequencing, to further identify the genes and molecular pathways within the innate immune system of BP patients. Such insights may help uncover key events that trigger autoimmunity in genetically predisposed individuals with BP.
